# Unbounded number line estimation as a measure of numerical estimation

**DOI:** 10.1371/journal.pone.0213102

**Published:** 2019-03-14

**Authors:** Regina Miriam Reinert, Matthias Hartmann, Stefan Huber, Korbinian Moeller

**Affiliations:** 1 Benoit Consulting AG, Bern, Switzerland; 2 University of Bern, Department of Psychology, Bern, Switzerland; 3 Swiss Distance Learning University, Faculty of Psychology, Brig, Switzerland; 4 Leibniz-Institut für Wissensmedien, Tübingen, Germany; 5 Groz-Beckert KG, Albstadt, Germany; 6 LEAD Graduate School & Research Network, Tübingen, Germany; 7 University of Tübingen, Department of Psychology, Tübingen, Germany; French National Center for Scientific Research (CNRS) & University of Lyon, FRANCE

## Abstract

Number magnitude estimation has been investigated over the last decades using different tasks including non-symbolic numerosity but also number line estimation tasks. Recently, a bi-directional mapping process was suggested for numerosity estimation accounting for underestimation in a perception version of the task (i.e., indicating the number of non-symbolic dots in a set) and overestimation in the corresponding production task (i.e., produce the number of dots indicated by a symbolic number). In the present study, we evaluated the generalizability of these estimation biases in perception and production tasks to bounded and unbounded number line estimation. Importantly, target numbers were underestimated/overestimated by participants in the perception/production version of numerosity estimation as well as unbounded number line estimation. However, this pattern was reversed for bounded number line estimation. Thereby, the present data indicate a conceptual similarity of unbounded number line estimation and the established non-symbolic numerosity estimation task as a measure of numerical estimation. Accordingly, this corroborates the notion that unbounded number line estimation may reflect a purer measure of number magnitude representation than the bounded task version. Furthermore, our findings strengthen the bi-directional mapping hypothesis for numerical estimation by providing evidence for its generalizability to unbounded number line estimation for the first time.

## Introduction

Magnitude estimation tasks are typically employed to investigate numerical cognition. On the one hand, non-symbolic stimuli like collections of dots or sequences of sounds are used to assess the underlying representation of number magnitude. Usually, participants have to estimate their numerosity producing symbolic outputs such as Arabic or oral verbal numerals (e.g., [1],[2]). On the other hand, the (spatial) representation of number magnitude is often investigated using symbolic stimuli in tasks such as number line estimation (e.g., [3]). So far, however, similarities and differences in performance patterns for non-symbolic numerosity estimation and number line estimation have hardly been investigated. Therefore, the current study set off to evaluate whether results on non-symbolic numerosity estimation (e.g., [4]) can be generalized to bounded (e.g., [3]) as well as unbounded number line estimation (e.g., [5]). Generalizable patterns of results would provide further evidence that bounded and/or unbounded number line estimation indeed rely on number magnitude estimation processes. In the following, we will first elaborate on the specifics of number line estimation and non-symbolic numerosity estimation before outlining the details of the present study.

### Number line estimation tasks

The traditional version of this task is the bounded number line estimation task in which participants have to indicate the spatial position of a target number (e.g., 45) on a number line with a given start and endpoint (e.g., 0 to 100; e.g. [3],[6–10]). The observed estimation pattern is then used to infer on the nature of the underlying representation of number magnitude.

However, it has been controversially debated in recent years whether this task indeed allows for inferences on the spatial layout of the underlying mental number line representation (e.g., [3]) or rather task-specific strategies that participants apply while they solve the task [11–13]. Barth and Paladino [14] were the first to argue that the observed estimation pattern in this task may not reflect pure numerical estimation but the use of proportion-judgement strategies (see also [5],[13],[15]). Support for the assumption of the use of such task-specific strategies comes from the observation that participants’ estimates are biased towards specific reference points (e.g., start and endpoint of the scale as well as the middle) with estimations being relatively more accurate near these reference points.

Recently, Cohen and Blanc-Goldhammer [5] presented a new unbounded version of the number line estimation task and argued that this version provides a purer measurement of numerical estimation than the original bounded version. In this task version, only the start point and a scaling unit, but no endpoint of the number line is given. The observed estimation patterns led the authors to the conclusion that the bounded number line estimation task may be an invalid measure of number magnitude representation. Importantly, the results observed in this unbounded number line estimation task differed from the typical error pattern found in the bounded version of the task: Instead of estimations being more accurate around reference points, the authors found an error pattern that was consistent with scalar variance (see [16–19]), this means that estimation errors increased linearly with the size of the target number (see also [20]). Interestingly, this nicely reflects the pattern of results usually observed in estimation tasks in which, for instance, the number of dots in a given set has to be indicated (e.g., [4],[21],[22]).

So far, however, this potential conceptual similarity between unbounded number line estimation and the estimation of the magnitude of non-symbolic stimuli has not been investigated yet. Instead, the argument that unbounded number line estimation reflects a purer measure of numerical estimation has primarily been made based on comparisons of estimation performance in unbounded and bounded number line estimation. This seems surprising because non-symbolic numerosity estimation tasks are generally agreed to reflect a reliable measure of numerical estimation. Therefore, the current study set out to systematically evaluate similarities and differences between non-symbolic estimation and unbounded as well as bounded number line estimation. We expected a closer association of non-symbolic estimation with unbounded than with bounded number line estimation.

### Numerosity estimation

To do so, we followed the suggestion of Crollen, Castronovo and Seron ([4]; see also [23] for a taxonomy of paradigms of studies on magnitude estimation) and specifically investigated differences between production and perception type of the respective estimation tasks. For tasks using numbers of dots as non-symbolic targets, Crollen et al. [4] showed opposing biases for the different types of the tasks (i.e., production and perception version). In their numerosity perception task, participants estimated the numerosity of collections of dots and expressed their estimates via symbolic Arabic numerals. In contrast, in their numerosity production task, symbolic stimuli in terms of an Arabic number were presented to participants who then had to estimate the denoted magnitude by non-symbolic output, this means, by adjusting the numerosity of a dot pattern so that it reflected the magnitude of the presented Arabic numeral. Depending on task type, the authors observed opposing patterns of performance: The numerosity of a dot set was systematically underestimated in the perception task, whereas the numerosity produced in the production task was overestimated significantly (see also [21]). The authors accounted for these systematic biases of under- and overestimation by the bi-directional mapping-hypothesis (see also [24–27]) that is based on three assumptions: (1) There are different numerical representations. In the present context, the first assumption refers to the differentiation between symbolic (e.g., 3) and non-symbolic (e.g., •••) representations of numerical magnitude. (2) Transcoding routes between these representations are assumed that allow for translating magnitude information from one representation to the other (i.e., symbolic to non-symbolic and non-symbolic to symbolic). Moreover, it is supposed that (3) precision differs between different numerical representations. The non-symbolic representation is assumed to be less precise as it is logarithmically compressed resulting in decreasing differences between adjacent numbers with increasing magnitude (e.g., [28–30]).

**Fig 1:**
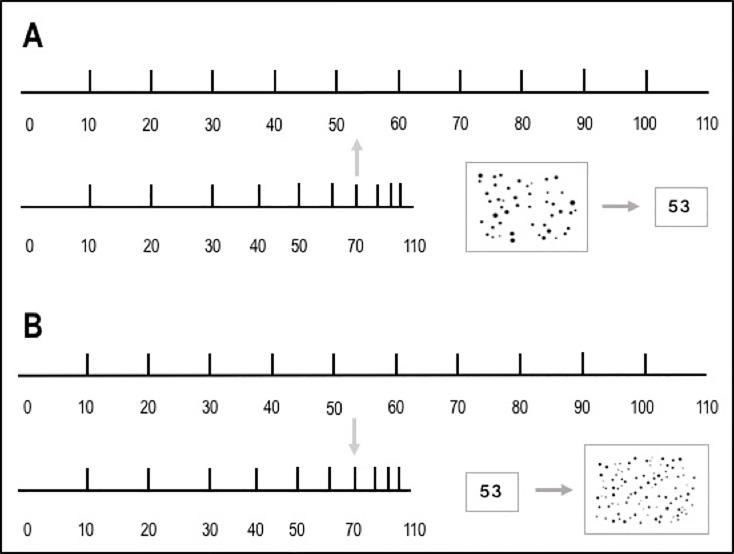
Schematic illustration of Crollen et al.’s ([4], p. 41) bi-directional mapping processes (in gray arrows): Panel (A) shows the assumed estimation process in a perception task requiring estimation from the logarithmically compressed representation of non-symbolic numerosity onto the linearly spaced representation of symbolic number magnitude which leads to underestimation. Panel (B) depicts the estimation process in the production task, in which linearly spaced symbolic number magnitude representations have to be mapped onto the compressed representation of non-symbolic numerosity, hence, leading to overestimation.

Based on these three assumptions, the opposing biases observed in numerosity perception and production in terms of under- and overestimation are assumed to stem from biases in transcoding between symbolic representations and their corresponding analogue (non-symbolic) magnitude representations. In the perception task, the numerical estimation process goes from a position on the logarithmically compressed representation of non-symbolic magnitudes to its associated linear symbolic numerical representation. Accordingly, the subjective magnitude is constantly larger compared to the objective one, and the perceived numerosity is therefore underestimated by participants. In contrast, in the production task, the mapping process starts from a position on the linear symbolic numerical representation and goes to its corresponding analogue but logarithmically compressed magnitude representation on the subjective non-symbolic number line. In this case, the objective magnitude is smaller than the subjective one. Hence, this leads to an overestimation of the target magnitude (see Fig 1A).

Interestingly, even though number line estimation is argued to capture processes of magnitude estimation, there is only very few research on number line estimation considering both directions of the mapping. To the best of our knowledge, there are so far only two studies directly comparing a perception version (position-to-number) with the more commonly used production version (number-to-position) of the bounded number line estimation task. First, Siegler and Opfer [3] observed that estimates of second graders in US produced linear estimation patterns on a 0 to 100 scale in the production version of the task, but logarithmic one on the 0 to 1000 scale. For the perception version of the task, the authors found that children’s estimation patterns were exponential in nature. In contrast, in older students as well as adults, results followed a linear pattern with no clear indication of under- or overestimation.

Additionally, Slusser and Barth [27] found relatively consistent estimation accuracy across both task versions as well as patterns of under- and overestimation specific task version. Additionally, proportion estimation models offered a more appropriate explanation of participants’ performance than linear, logarithmic or exponential models and consistent results of estimation bias remain on both bounded task versions over the course of development. These findings indicate that the different task versions of the bounded number line estimation task induce people to apply different solution strategies calling into question whether this task is a valid measure of mental number representation. Besides, only little work has been done applying the perception version of a number line estimation task (cf. [15], [31]).

For unbounded number line estimation, there is currently no study investigating similarities and differences between the production and perception version of the task. In fact, we are not aware that the latter was ever used in research before. Furthermore, there is currently no study evaluating the conceptual similarity of bounded and unbounded number line estimation to with analogue (non-symbolic) numerosity estimation.

### The present study

In the present study, we therefore aimed at investigating the conceptual similarity of unbounded and bounded number line estimation with non-symbolic numerosity estimation. In line with previous results, we generally expected to replicate the previous findings of Crollen and colleagues [4] in terms of underestimation in our numerosity perception task and overestimation in our numerosity production task.

Furthermore, in line with the argument that unbounded number line estimation reflects a measure of numerical estimation (e.g., [5]), we hypothesized that estimation performance in the two versions of the unbounded number line estimation task should follow the pattern of under- and overestimation observed in the perception and production version of the numerosity estimation task. In the perception version of unbounded number line estimation, participants should systematically underestimate numerical magnitudes whereas we expected them to overestimate numerical magnitude in the production version. So far, a perception version of this task has never been described in the literature, only the production version.

The single unit distance at the origin of the number line as well as the hatch mark at the position to be estimated in the perception task of the unbounded number line estimation task are assumed to activate the non-symbolic, analogue representation of numbers. When estimating the corresponding Arabic number reflecting the spatial position on the number line, participants have to transcode from the logarithmically compressed analogue to the symbolic linear Arabic representation. Accordingly, corresponding values of the target number are always smaller and participants are expected to underestimate the respective numerical magnitude. The other way round, in the production task, we expect that the magnitude of the spatial position of the target number on the number line is overestimated by participants as the numerical estimation process is based on symbolic to non-symbolic mapping. In particular, in this version of the task, the target number is presented as an Arabic number that has to be transcoded into an analogue representation of numerosity. Therefore, the estimation process starts from a position on the symbolic linear numerical representation to its associated magnitude on the logarithmically compressed subjective number line. As such, objective magnitude seems smaller than its subjective counterpart and participants should overestimate the respective magnitude. Observation of such a result pattern would provide additional evidence for the assumption that the unbounded number line estimation task reflects a more unbiased measure of the mental number line representation as compared to the bounded number line estimation task.

Because bounded number line estimation was supposed to entail proportion-based judgments, we expected a more task-specific result pattern. With regard to over- and underestimation in the production and perception task version Siegler and Opfer ([3], see also [27]) found inconsistent result patterns for their children and adult participants. Therefore, we had no specific expectations with respect to the pattern of over- and underestimation in bounded number line estimation.

**Fig 2:**
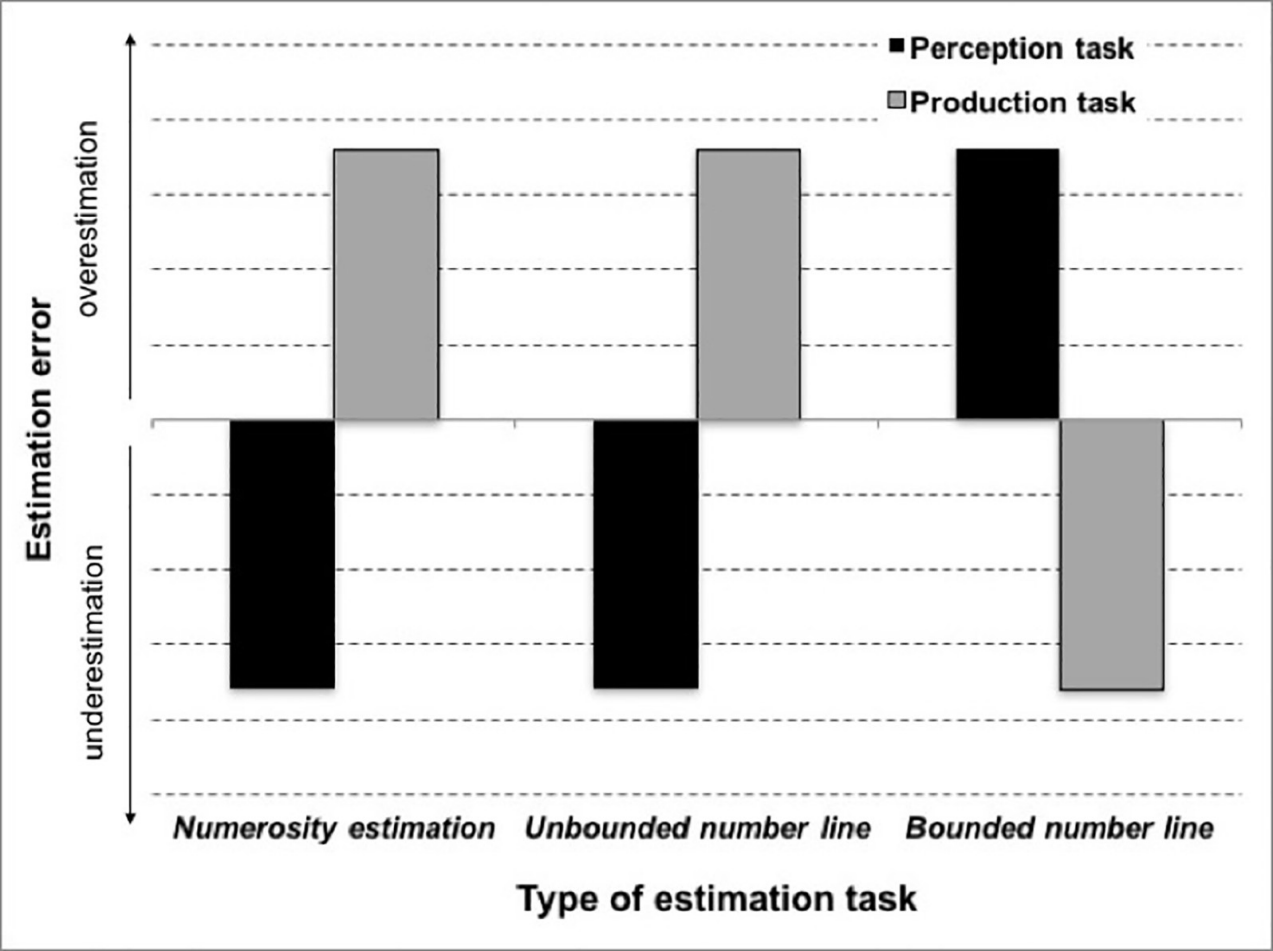
Expected error patterns in the three different types of estimation tasks. We hypothesized that error patterns of numerosity estimation and unbounded number line estimation should be identical, whereas the error pattern for bounded number line estimation should be reversed.

In sum, considering the bi-directional mapping hypothesis, we put forth the following hypotheses concerning the estimation patterns of performance in the different tasks (see Fig 2 for the expected error pattern): (1) a replication of the result pattern of Crollen and colleagues [4] in non-symbolic numerosity estimation with underestimation in the perception and overestimation in the production version of the task. (2) Because unbounded number line estimation has been argued to reflect a purer measure of numerical estimation, we analogously expected underestimation in the perception and overestimation in the production version of the task. In contrast, for (3) bounded number line estimation we did not have any specific expectation in both versions of the task.

Our study addressed this issue by a systematic comparison between estimation patterns from both bounded and unbounded number line estimation with analogue numerosity estimation. We expected that the systematic pattern of under- and overestimation in the numerosity estimation task should generalize to unbounded but not bounded number line estimation. In this case, our data would provide converging evidence for the claim that unbounded number line estimation is indeed a purer measure of number magnitude estimation from a new, complementary perspective never been taken before.

## Methods

### Participants

A total of 75 German-speaking students of the University of Bern (21 males; 10 left-handers) participated voluntarily in the experiment for course credits. The average age was 23.6 years (*SD =* 4.4 years; *range =* 19–39 years). All participants reported normal or corrected-to-normal vision. Additionally, all participants signed an informed consent form prior to the study, which was approved by the local ethics committee of the University of Bern (Nr. 2017-02-00008).

### Stimuli and procedure

Participants were assessed individually with a battery of tasks which were instructed separately. For all tasks, stimuli were presented as pictures on a laptop with a 15´´ screen with a resolution of 1,024 x 768 pixels. Each task consisted of two types of estimation: perception and production of numerosities. The perception version of each task was always administered first to avoid that the maximal number of the numerosity presented in the production task made participants anticipate the maximum magnitude in the production task. Moreover, participants received no information about the range of numerosities being presented and no feedback was provided. Items were presented in randomized order for each participant individually.

Tasks were administered in the following order: First, two different numerosity perception tasks were presented to participants whereas the order of both versions was counterbalanced. Half of the participants started with a version in which all presented dots had the same size whereas the other half began with the version in which overall area covered by the presented dots was matched (see next section for details). These tasks were directly followed by the numerosity production task. Second, perception and production versions of the unbounded number line estimation task were administered prior to the two respective versions of the bounded number line estimation tasks. Thereby, the task which explicitly defines a number range (i.e. bounded number line estimation) was administered last to avoid participants building up expectations about the number ranges used in the tasks. For both, numerosity estimation as well as unbounded number line estimation the upper bound of the number range covered by the tasks is not specified to participants, and thus they may not use a given upper bound as an additional reference point. As the bounded number line estimation task explicitly defines a number range by its upper bound, it was conducted last to prevent participants to assume a similar number range for numerosity and unbounded number line estimation.

Two additional control tasks were given last: one task in which participants had to estimate proportions of areas like triangles, circles, squares and rectangles and the Berlin Numeracy Task [32]. These were administered to investigate a research question different from the one described in the present study. Therefore, results for these two tasks will not be considered in the present study. In sum, the experiment took approximately 45 min.

#### Numerosity estimation

Twenty-four different numerosities were used to create the target sets of dots that ranged from 30 to 100. Two different target sets were developed: one for the two *perception tasks* (30, 34, 39, 41, 43, 46, 48, 52, 55, 57, 60, 64, 65, 69, 70, 75, 78, 82, 83, 86, 87, 91, 94, 99), and an additional one for the *production task* (31, 35, 38, 40, 42, 47, 49, 53, 54, 56, 62, 63, 66, 67, 71, 76, 77, 80, 84, 85, 89, 92, 93, 98). Target numbers were chosen to have the same mean problem size for the overall number range as well as numbers within each decade (i.e. 31–39, 41–49, etc.). Furthermore, we developed two different versions of stimuli for the perception task to control for perceptive parameters such as total occupied area and dot size. In one stimuli set, all dots had the same size and therefore the area covered by the array increased with increasing numerosity. In the other set, the sum of the area of all the dots on the screen was kept constant.

Each trial in the *numerosity perception task* started with a centrally presented black fixation cross presented for 1,000 ms against a white background followed by a pattern of dots that was flashed on the screen for 250 ms. Afterwards, the sign “=“ was displayed and replaced after 500 ms by the Arabic numeral “1”. Participants’ task was to indicate as quickly and accurately as possible the numerosity of presented dots as an Arabic number. To give their answers, they had to scroll the mouse wheel up through the sequence of Arabic numbers (or down to go back to correct the answer) and press the “Enter” button to finish their estimation. No information about the number range and feedback concerning their performance were provided.

In the *numerosity production task*, participants were required to produce a dot pattern that was equivalent to the presented Arabic number magnitude. Each trial started with the presentation of a black fixation cross (for 1,000 ms) in the middle of the screen against a white background, followed by the two-digit target Arabic numeral for 250 ms. Then, the sign “=“ was displayed (500 ms) and the production phase began with a single dot on the screen at a randomly determined position. Participants then had to start dots production by scrolling the mouse wheel up to increase the number of dots that appear on the screen and down to decrease the number of dots. When they had the impression that the numerosity of dots corresponded to the requested number, they pressed the “Enter” button. The maximum number of dots that participants could produce was limited to 254 (cf. Experiment 2 of [4]).

#### Number line estimation

**Fig 3:**
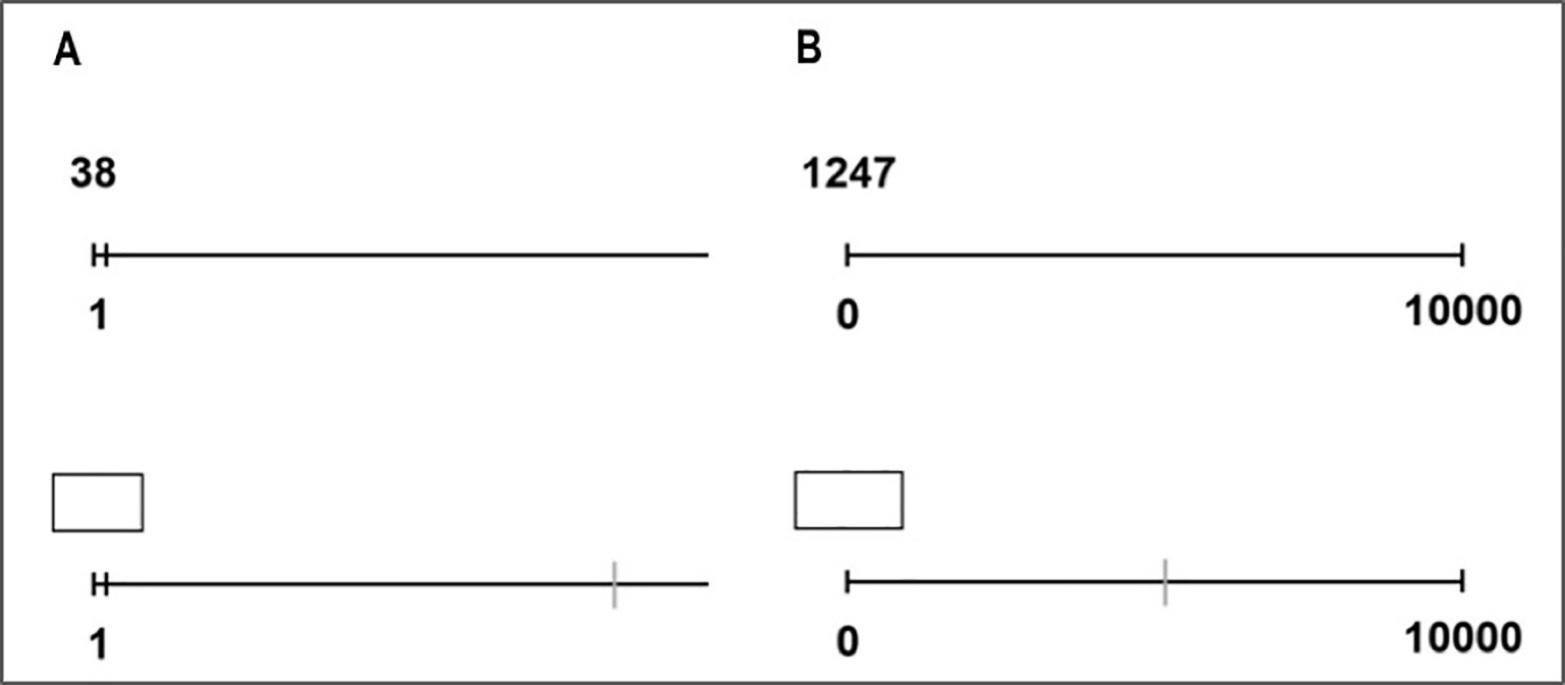
Schematic illustration of an example of the (A) unbounded and (B) bounded number line estimation. The production tasks are displayed in the upper row, the perception tasks in the lower row.

In the production version of both unbounded and bounded number line estimation tasks, participants were instructed to indicate as accurately and as fast as possible the spatial position of the given target number on the number line using the mouse to click at the estimated position (upper panels of Fig 3A and 3B). In the perception version of the tasks, participants had to insert the Arabic number specifying the spatial location already indicated on the number line by using the keyboard. The number lines as well as target numbers were presented in black colour against a white background (see Fig 3).

#### Unbounded number line estimation task

A total of twenty-four items were used as target sets ranging from 2 to 49, one for the *perception task* (3, 4, 7, 8, 11, 12,15, 16, 19, 20, 23, 24, 27, 28, 31, 32, 35, 36, 39, 40, 43, 44, 47, 48) and another for the *production task* (2, 5, 6, 9, 10, 13, 14, 17, 18, 21, 22, 25, 26, 29, 30, 33, 34, 37, 38, 41, 42, 45, 46, 49). Here, too, target numbers were chosen to have the same mean problem size regarding both numbers within each decade as well as the overall number range covered. Each number line was a horizontal line with a physical length of 18 cm. Only the start-point together with the predefined unit of 1 having a length of 0.3 cm was presented (see Fig 3, Panel A). We used a smaller number range for unbounded number line estimation as there is no evidence that number range influences participants’ estimation patterns (i.e., increasing error variability with increasing target numbers). In fact, estimation patterns were virtually identical across smaller and larger ranges (e.g., 25 in [5]; 49 in [20]; 400 in [33]; 1,000 in [34]).

In the *perception task*, a response box was displayed above the start-point and the position to be estimated was marked with a blue vertical line. Participants had to insert the corresponding Arabic number reflected by the spatial position on the number line using the number keys on the keyboard. To complete the estimation, they had to press the “Enter” button and then, the next trial appeared at a random position on the screen to prevent participants from using external reference points.

In the *production task*, target numbers were displayed above the start point of the number line (see Fig 3, lower chart) at a position on the screen randomly varying from trial to trial. The blue vertical line which reflected the mouse curser always appeared in the centre of the screen with a vertical length of 1.4 cm. To give their responses, participants had to mark the estimated spatial position of the respective target number by moving the blue line mouse curser there and click on the felt button of the mouse.

#### Bounded number line estimation task

The bounded number line estimation task covered the number range from 0 to 10.000 using the target numbers (162, 453, 820, 1027, 1644, 2341, 2660, 3221, 3786, 4259, 4575, 4808, 5128, 5420, 5827, 6390, 6915, 7237, 7682, 8406, 8753, 9049, 9561, 9876) for the *perception task* and an additional target set (124, 439, 951, 1247, 1594, 2318, 2763, 3085, 3610, 4173, 4580, 4872, 5192, 5425, 5741, 6214, 6770, 7340, 7659, 8356, 8973,9180, 9547, 9838) for the *production task*. These were selected with a slight oversampling at the midpoint 5.000 as well as the start- and endpoint (0 and 10.000) as reference marks. Start- and endpoint were always displayed at the same position on the screen and the physical length of the number line was 18 cm.

In the *perception task*, a response box was presented above the start-point and the spatial location of the target number was marked with a blue vertical line. Participants had to insert the Arabic numbers reflecting the position on the number line using the number keys of the keyboard (see Fig 3, Panel B). To give finalize their answers, they had to press the “Enter” button. Then, the next trial appeared.

In the *production task*, participants were required to indicate the spatial position of a given number on an empty number line with specified start- and endpoint. Therefore, they were instructed to mark their estimated position of the respective target number with the blue vertical line always appearing in the left lower corner on the number line.

### Analyses

As preliminary analyses indicated that mean estimates as well as their standard deviations were not normally distributed in all tasks, we used the median as a measure of central tendency of the relative estimation errors. For all magnitude estimation tasks, the mean percent relative estimation error [REE = (estimation number–target number)/number range of the task * 100] served as dependent variable, this means that we standardized participants’ estimation errors on the number range of the respective task to increase comparability of results. Please note that we were not interested in comparing the unstandardized magnitude of estimation errors across the different task versions. Instead, we were specifically interested in the pattern of over- and underestimation for perception and production versions of the three tasks, which should be reflected in REE.

**Fig 4:**
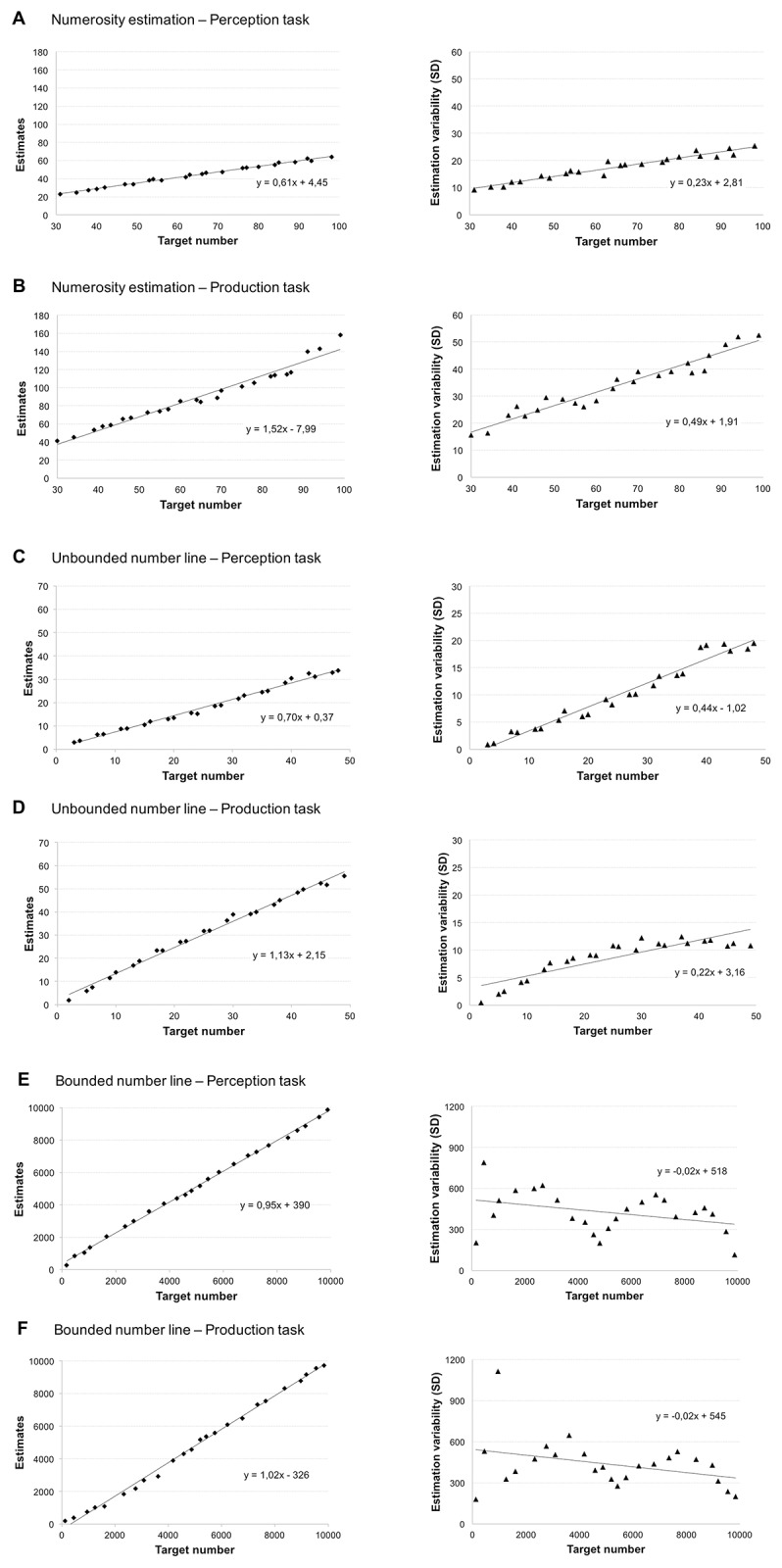
Estimation patterns (mean estimates across all participants, left charts) and estimation error variability (SD of REE, right charts) for numerosity estimation (Panels A + B), unbounded number line estimation (Panels C + D) and bounded number line estimation (Panels E + F).

Number ranges considered were 1 to 10,000 for bounded and 2 to 49 in the unbounded number line estimation task as well as 30 to 99 in the numerosity estimation task. Please note that to evaluate patterns of over- and underestimation we used the *relative* estimation error as dependent variable and not the more often used absolute estimation error (e.g., [3]). Accordingly, a REE of zero would reflect accurate estimations, whereas negative REE indicate underestimation and positive REE overestimation of the target numbers. Furthermore, in case the sphericity assumption of the ANOVA (analysis of variance) was violated, the Greenhouse-Geisser coefficient (GG) is given to adjust the degrees of freedom. An overview of participants’ estimation patterns (left charts) and error variability (right charts) is given in Fig 4.

## Results

In a first step, individual trials that differed more than ± 4 standard deviations from the overall mean estimates were excluded from the analysis (0.25% of the data). Moreover, we used the average mean of both versions of the perception numerosity estimation task as dependent variable for the ANOVA because the correlation between REE in both versions was *r*(75) *=* .89, *p <* .001, and therefore sufficiently high to pool the respective means.

**Fig 5:**
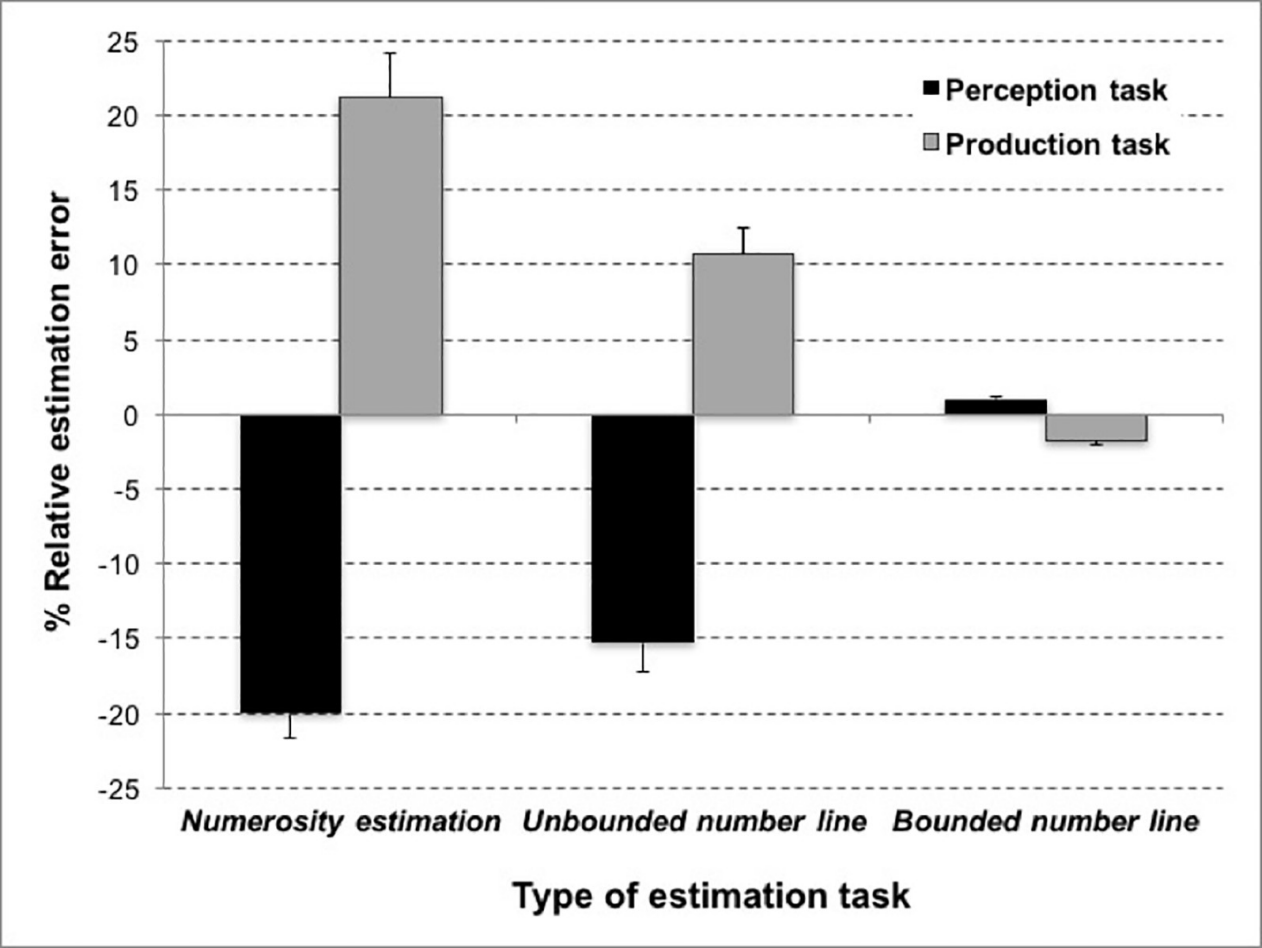
Mean percent REE for the three magnitude estimation tasks separated for perception and production task type. Error bars reflect 1 Standard Error of the Mean (SEM).

To examine the performance patterns in the different estimation tasks, we ran a 3 x 2 repeated-measures ANOVA with the factors *type of estimation* (numerosity estimation, unbounded vs. bounded number line estimation task) and the *task type* (perception vs. production; see Fig 5). This analysis revealed a significant main effect of task type, *F*(1, 74) = 167.80, *p <* .001, *η*^*2*^_*p*_ *=* .694, indicating that participants showed systematic underestimation in perception and overestimation in production tasks (*M*_*perception*_ *=* -11.44% vs. *M*_*production*_ *=* 10.04% REE). The main effect for type of estimation *F*(2, 148) = 2.44, *p =* .104, *η*^*2*^*p =* .032, *GG* = .783, was not significant, reflecting that there were no significant differences in accuracy of participants’ estimation patterns between the three estimation tasks (*M*_*numerosity estimation*_ *=* .61 vs. *M*_*bounded*_ *=* -.40 vs. *M*_*unbounded*_ *=* -2.30).

Most importantly, we observed that the main effect of task type was qualified by the significant interaction between the factors type of estimation and task type *F(*2, 148) = 59.03, *p <* .001, *η*^2^*p =* .444, *GG =* .782. The interaction indicated that, in line with our expectations, target numbers were underestimated in the perception version of the numerosity estimation task (*M*_*perception*_ = -20.01% vs. *M*_*production*_ = 21.24% REE) as well as the unbounded number line estimation task (*M*_*perception*_ = -15.32% vs. *M*_*production*_ *=* 10.72% REE) whereas participants overestimated target numbers in the production version of these two tasks. However, this pattern was reversed for bounded number line estimation. Here, participants overestimated target numbers in the perception version and underestimated then in the production version of this task (*M*_*perception*_ *=* 1.02% vs. *M*_*production*_ *=* -1.83% REE). Testing simple effects using Bonferroni-Holm corrected *t*-tests revealed that estimation accuracy differed significantly between perception and production versions of all tasks (numerosity estimation: *t*(74) = 10.35, *p <* .001; bounded number line estimation: *t(*74) = 9.66, *p <* .001; and unbounded number line estimation: *t*(74) = 8.55, *p <* .001.

## Discussion

The present study set off to systematically investigate similarities and differences between non-symbolic numerosity estimation (e.g., [4]) and bounded (e.g., [3]) as well as unbounded number line estimation (e.g., [5]). In particular, the aim of our study was to examine the generalizability of patterns of underestimation in the perception and overestimation in the production version of non-symbolic numerosity estimation to bounded and unbounded number line estimation. This approach seems promising to evaluate whether unbounded number line estimation is more similar to non-symbolic estimation and thus reflects a purer measure of magnitude representation. In the following, we will first discuss the results with respect to unbounded number line estimation as a measure of number magnitude representation before elaborating on the broader implications of these findings for the bi-directional mapping hypothesis.

### Unbounded number line estimation as a purer measure of number magnitude representation

We expected to replicate the result pattern of underestimation in the perception and overestimation in the production version non-symbolic numerosity estimation [4]. Furthermore, we analogously hypothesized the same pattern of estimation errors for the perception and production version of unbounded number line estimation. The current data corroborated these two hypotheses by revealing that systematic estimation biases were identical for non-symbolic numerosity and unbounded number line estimation. For both estimation tasks, we observed that participants systematically underestimated target numbers in the perception, and overestimated them in the production version.

Importantly, our data provide additional evidence for the argument of Cohen and Blanc-Goldhammer [5] that unbounded number line estimation might be a purer measure of the (spatial) representation of number magnitude than bounded number line estimation (see also [12],[14]). So far, the argument that unbounded number line estimation represents a more valid measure of number magnitude representation originally came from comparisons of estimation performance in bounded and unbounded number line estimation. Here, we showed its similarity with non-symbolic numerosity estimation that is commonly agreed on to constitute a reliable measure of number magnitude representation.

Additionally, the observation of a reversed estimation pattern of overestimation in the perception and under estimation in the production version for bounded number line estimation further corroborates this interpretation. These findings are in line with our expectations and seems to reflect previous findings of Siegler and Opfer [3] who observed a systematic pattern of overestimation in the perception task, at least in children. Additionally, for the production version of the task Booth and Siegler [7] observed evidence for underestimation in the production version of the task. This pattern of under- and overestimation clearly differed for that found for numerosity estimation and unbounded number line estimation. Therefore, these data provide converging evidence for the notion that bounded number line estimation may not only measure numerical estimation but also task-specific strategies (see [12]–[14]).

In sum, our results seem to substantiate claims that unbounded number line estimation may be more suitable to draw inferences on adults´ (spatial) representation of number magnitude (see also [10], [35]). Importantly, however, these data are not only relevant for our understanding of unbounded number line estimation but also for the bi-directional mapping hypothesis on non-symbolic magnitude estimation.

### Further evidence for the bi-directional mapping hypothesis

For the first time, we investigated perception and production versions for all non-symbolic, unbounded as well as bounded estimation tasks. As expected, we observed that the characteristic pattern of overestimation in the production and underestimation in the perception version of numerosity estimation [4] as well as the unbounded number line estimation task. Importantly, this is in line with the postulates of the model on the mapping between symbolic and non-symbolic representations proposed by Izard and Dehaene [36]. They suggested different mapping processes from non-symbolic input to symbolic output and symbolic input to non-symbolic output, leading participants to systematically under- and overestimate the respective target magnitudes, respectively.

In the perception version of numerosity estimation as well as unbounded number line estimation, the estimation process goes from the logarithmically compressed representation of non-symbolic numerosity to the linearly spaced representation of symbolic number magnitude resulting in the observed underestimation. In the perception version of unbounded number line estimation, the unit distance given at the origin of the number line as well as the hatch mark at the position to be estimated activated a non-symbolic, analogue representation of the respective target numbers. When estimating the corresponding Arabic number based on predefined spatial position on the number line, participants had to transcode from the non-symbolic analogue into the symbolic Arabic number form. Consequently, the corresponding values of the target numbers are always smaller and participants underestimate numerical magnitude. In contrast, in the production version of unbounded number line estimation, a linearly spaced representations of symbolic number magnitude had to be mapped onto the logarithmically compressed representation of non-symbolic numerosity, which, in turn, led to the observed pattern of overestimation (see Fig 1). These findings for unbounded number line estimation further strengthen the bi-directional mapping hypothesis [21] by providing convergent evidence from perception and production versions for the first time.

### Limitations and perspectives

There are aspects to keep in mind when interpreting the current results. On a methodological level it should be acknowledged that to get an accurate measure of estimation errors in unbounded number line estimation, it is critical to allow enough room for participants to express errors. In the current task, the number line had a length of 50 units and target numbers up to 49 had to be estimated. Therefore, the end of the number line may have acted as a boundary that participants will likely not extend their answer past. This might have influenced estimation errors for target numbers approaching 49. However, the overall pattern of estimation errors we observed in the unbounded task was more or less identical to the patterns observed in previous studies (e.g., [5],[35]). Therefore, we are confident that this should not have biased results. Nevertheless, it may be desirable for future studies to allow more space between the largest target number and the end of the number line so that implicit boundaries are so far beyond the participant's likely response that they will not influence it.

On the theoretical level, it is important to note that the bi-directional mapping hypothesis implicitly assumes a logarithmically compressed non-symbolic representation of number magnitude representation in comparison to a linear one for symbolic magnitudes. In this context, it should be considered that there is a long-lasting scientific debate about the layout of number magnitude representations (i.e., logarithmically compressed vs. linear, e.g., [37]; see also [17], [38]). However, it was not at the heart of this study to evaluate this. Instead, we only considered the predictions of the bi-directional mapping hypothesis, which reflect specific assumptions on logarithmic non-symbolic and linear symbolic magnitude representations. As such, the observation of identical patterns of over and underestimation in the perception vs. production version of numerosity and unbounded number line estimation might imply a similar logarithmic layout of the representation of non-symbolic magnitude in unbounded number line estimation. However, further research is needed addressing this question more specifically.

Finally, as also noted by Crollen and colleagues [4], the investigation of the mapping process between symbolic and non-symbolic magnitude representations is only in its early stages with very few research directly addressing this question. More precise and general conclusions will become possible when taking a closer look at the development of the numerical mapping abilities in children (see also [35],[39],[40]). Future studies should therefore further investigate the model of bi-directional mapping by exploring data of children. It would be interesting whether the same pattern of under- and overestimation would be replicated in such a sample.

## Conclusions

Taken together, to the best of our knowledge, the present study is the first to systematically assess different types of estimation tasks (i.e., numerosity, as well as bounded and unbounded number line estimation) in both their perception and production version in adults to evaluate similarities and differences between these tasks. We replicated the pattern of systematic biases of under- and overestimation for numerosity estimation and also found that this pattern generalized to unbounded but not bounded number line estimation. Therefore, our results indicated conceptual similarity of unbounded number line and non-symbolic numerosity estimation. As such, these findings provide converging evidence from results of an established magnitude estimation task that unbounded number line estimation might be a purer and more valid measure of (spatial) number magnitude representation as compared to bounded number line estimation.

## Supporting information

S1 Dataset SPSS file of the data. (SAV)

S2 Dataset Txt file of the data. (TXT)

S1 File Description of variables (Readme). (XLSX)
